# Anti-Müllerian hormone level may predict successful pregnancy after adenomyomectomy in patients with infertility due to adenomyosis

**DOI:** 10.1097/MD.0000000000026075

**Published:** 2021-05-28

**Authors:** Seyeon Won, Ji Young Hwang, Nara Lee, Miseon Kim, Mi Kyoung Kim, Mi-La Kim, Bo Seong Yun, Seok Ju Seong, Yong Wook Jung

**Affiliations:** a Department of Obstetrics and Gynecology, CHA Gangnam Medical Center, CHA University College of Medicine; b Department of Obstetrics and Gynecology, Fertility Center of CHA Gangnam Medical Center, CHA University, Seoul, Republic of Korea.

**Keywords:** adenomyosis, anti-Müllerian hormone, fertility, ovarian reserve

## Abstract

We aimed to determine clinical factors predicting successful pregnancy by comparing pregnancy failure and success groups after adenomyomectomy. Additionally, we analyzed fertility outcomes after adenomyomectomy.

The medical records of 43 patients who had undergone adenomyomectomy and received in vitro fertilization treatment from 2017 to 2020 were retrospectively reviewed. Patients were divided into pregnancy failure (n = 28) and pregnancy success (n = 15) groups. Patients’ demographic factors were evaluated and compared between the groups.

The age of patients was higher (39.0 [32.0–45.0] vs. 37.0 [33.0–42.0] years, *P* = .006) whereas the level of anti-Müllerian hormone (anti-Müllerian hormone [AMH]; 0.54 [0.01–8.54] vs. 2.91 [0.34–7.92] ng/mL, *P* = .002) lower in the pregnancy failure group compared to the pregnancy success group. The operative time was longer (220.0 [68.0–440.0] vs. 175.0 [65.0–305.0] min, *P* = .048) while the estimated blood loss higher (750 [100–2500] vs. 500 [50–2000] mL, *P* = .016) in the pregnancy failure group compared to the pregnancy success group. No significant difference was observed in body mass index, symptoms, cancer antigen 125, preoperative uterine volume, or type of adenomyosis. In the multivariate analysis, age and AMH were significant predictive factors for successful pregnancy.

Ovarian reserve (age and AMH) and disease severity might be predictive factors for successful pregnancy in patients who have undergone adenomyomectomy. Adenomyomectomy should be considered for women desiring pregnancy and having appropriate ovarian reserve. Our results would be beneficial for patients and clinicians before deciding on adenomyomectomy. Larger prospective studies are required to confirm our findings.

## 1 Introduction

Adenomyosis is characterized by the presence of ectopic endometrial glandular and stromal tissues within the myometrium.^[1]^ Due to the lack of standard diagnostic criteria, the prevalence of adenomyosis varies from 5% to 70%.^[2–4]^ Adenomyosis is most commonly diagnosed in women in their 40s and 50s. Approximately 20% of cases of adenomyosis are diagnosed in women aged below 40 years.^[5]^

Although medical treatment, including nonsteroidal anti-inflammatory drugs, progesterone-releasing intrauterine devices, oral contraceptives, and gonadotropin-releasing hormone agonists, can be used to relieve symptoms, the definitive treatment for adenomyosis is hysterectomy.^[5,6]^ For patients desiring pregnancy, medical treatment or hysterectomy cannot be adopted, and adenomyomectomy, which is surgical removal of affected myometrial tissues, can be an alternative treatment option.^[7]^ Adenomyomectomy is a complicated surgical procedure involving removal of affected adenomyotic tissues, which is associated with abundant intraoperative bleeding. This complex surgical procedure is associated with frequent recurrence and spontaneous uterine rupture during subsequent pregnancy.^[8]^ However, the impact of adenomyosis on pregnancy outcomes is controversial. The effect of surgical removal of the adenomyotic tissues on the fertility outcome has not been clearly demonstrated. There is no consensus on the surgical indication of symptomatic adenomyosis for patients seeking fertility preservation. Literature on predictive factors for successful pregnancy postoperatively is scarce.^[9]^

The aim of the present study was to determine clinical factors predicting successful pregnancy through comparison between pregnancy failure and success groups after adenomyomectomy. In addition, we analyzed fertility outcomes after adenomyomectomy.

## 2 Methods

We retrospectively reviewed patient data at the department of obstetrics and gynecology, CHA Gangnam Medical Center, Seoul, Republic of Korea. The study was approved by the Institutional Review Board (IRB, GCI-18–27).

Women undergoing adenomyomectomy using robot-assisted surgery, laparoscopy, or laparotomy from January 2017 to April 2020 were included. Inclusion criteria were as follows:

1) age ≤ 45 years,
2) consent to undergo the surgery after being informed about the possibility of disease recurrence and operative blood loss, and
3) desire to be pregnant in the future. All patients underwent transvaginal ultrasonography and magnetic resonance imaging (MRI) for the preoperative diagnosis.

Adenomyosis was preoperatively diagnosed based on transvaginal ultrasonography when two or more of the following findings were present^[10]^:

(1) a mottled inhomogeneous myometrial texture with non-uniform echotexture;
(2) a globular uterus;
(3) scattered cystic spaces throughout the myometrium; and
(4) a “shaggy” indistinct endometrial stripe at the border.

When adenomyosis was suspected, MRI was performed. Adenomyosis was diagnosed in the presence of diffused or focal widening of the junctional zone (> 12 mm) forming an ill-defined area of low signal intensity on T2-weighted MRI.^[11,12]^

Possible causes of infertility other than adenomyosis were excluded after the following tests: hysterosalpingography (for tubal factor infertility), blood tests (for diabetes mellitus, chronic anovulatory disorder, hyperprolactinemia, and thyroid dysfunction), and semen analysis (for male factor infertility) preoperatively.^[13]^

Once adenomyosis was histologically confirmed postoperatively, patients were followed-up monthly for 3 months. The subsequent follow-ups were conducted every 3 to 6 months. Patients who desired pregnancy were allowed to try 3 months postoperatively.

The primary measured outcome was the comparison between pregnancy failure and success groups to identify clinical factors predicting successful pregnancy. Secondary outcomes were fertility outcomes after adenomyomectomy. The numeric pain rating scale was used to determine the severity of dysmenorrhea. We defined menorrhagia as menstrual bleeding limiting normal activities in women and causing anemia.^[14]^ The uterine size was measured using transvaginal ultrasonography. The uterine volume was calculated using the following formula: volume = 0.5233 × (anteroposterior diameter [cm]) × (longitudinal diameter [cm]) × (transverse diameter [cm]).^[15]^ We defined pregnancy success as the presence of fetal heartbeat at 6–7 weeks of gestation. Endometrial distortion was defined as distortion of the shape of the endometrium on preoperative MRI, as confirmed by the consensus of three authors (SYW, JYH, and YWJ). The number of pregnancy trials was defined as the number of embryos transferred.

### 2.1 Surgical procedures

Laparoscopic or robotic adenomyomectomy has been detailed in our previous study.^[16]^ Abdominal adenomyomectomy was performed in cases of diffuse adenomyosis. For abdominal adenomyomectomy, a Pfannenstiel skin incision was made to access the peritoneal cavity. Diluted vasopressin (4 units in 20 mL of saline) was injected into the subserosal surface and myometrium throughout the uterus. We made a vertical incision from the fundus to the anterior upper margin of the cervix. The endometrial cavity was opened sufficiently to permit the introduction of the index finger to identify the 1-cm margin of tissue above the endometrium. Adenomyosis was radically excised leaving a 1-cm margin of tissue above the endometrium and 1-cm margin of tissue below the serosal surface. The endometrium was repaired with 2–0 Vicryl sutures. The uterus was reconstructed with interrupted 2-0 Vicryl sutures without dead space to prevent hematoma formation. Uterine serosa with 1 cm of the underlying myometrium was sutured using continuous 1-0 Vicryl sutures. A hemovac drain was inserted into the pelvic cavity. Six gynecologic surgeons with extensive experience performed all surgeries.

### 2.2 Statistical analysis

The chi-squared test and Fisher's exact test were used to compare categorical variables. Non-parametric variables were compared using the Mann–Whitney U test. A *P-*value < 0.05 was considered to be statistically significant. Variables with *P*-values < 0.2 in the univariate analysis were included in the multivariable logistic regression model. Statistical analysis was performed using IBM SPSS Statistics version 24.0 (IBM Corp., Armonk, NY).

## 3 Results

**Figure 1:**
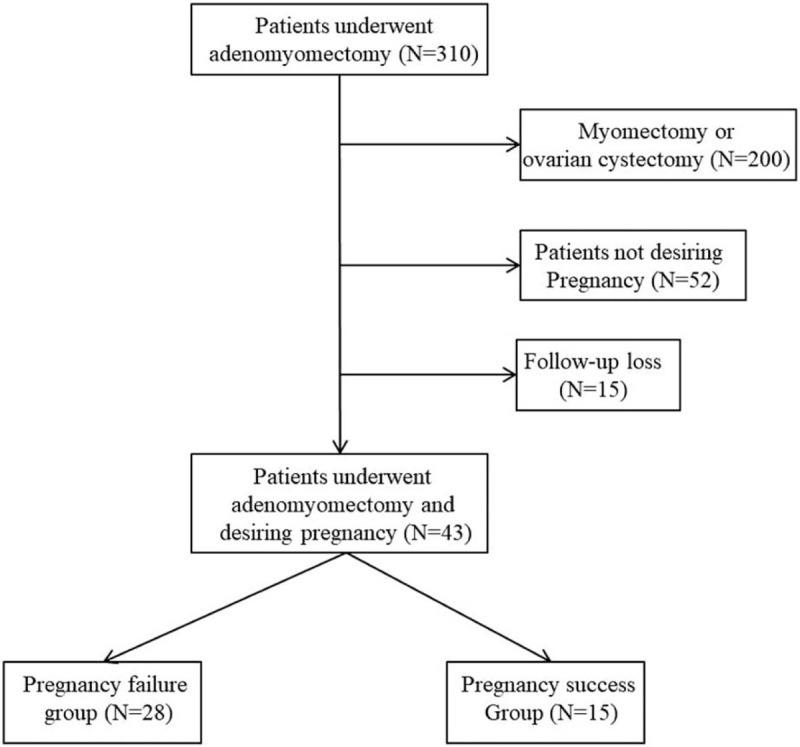
Flowchart of the study selection process.

A total of 310 women underwent adenomyomectomy. Among them, 200 patients were excluded because the major indications for the surgery in these patients were myoma and ovarian cyst. Fifteen patients were lost to follow-up, and 52 patients did not desire pregnancy postoperatively. Thus, 43 patients who had undergone adenomyomectomy and desired pregnancy postoperatively were included in the study. The included patients were divided into two groups: pregnancy failure (n = 28) and pregnancy success (n = 15; Fig. 1). Table 1 shows the baseline characteristics of the groups. The age of patients was higher (39.0 [32.0–46.0] vs 37.0 [33.0–42.0], *P* = .006) whereas the level of anti-Müllerian hormone (AMH) lower (0.54 [0.01–8.54] vs 2.91 [0.34–7.92] ng/mL, *P* = .002) in the pregnancy failure group compared to the pregnancy success group. The operative time was longer (220.0 [68.0–440.0] vs 175.0 [65.0–305.0] min, *P* = .048) while the estimated blood loss higher (750 [100–2500] vs 500 [50–2000] mL, *P* = .016) in the pregnancy failure group compared to the pregnancy success group. No statistically significant differences were observed in other variables between the groups.

**Table 1:** Baseline characteristics.

Demographics	Failure group (n = 28)	Success group (n = 15)	*P*
Age, yr	39.0 (32.0–45.0)	37.0 (33.0–42.0)	.006
BMI, kg/m^2^	22.0 (18.0–29.0)	21.8 (15.0–29.0)	.908
Gravida	0 (0–8)	1 (0–3)	.685
Parity	0 (0–1)	0 (0)	.295
Symptom			
Pain score (NRS)	8 (7–10, n = 20)	8.0 (1–10, n = 13)	.609
Menorrhagia (n)	24 (85.7%)	10 (66.7%)	.238
AMH, ng/mL	0.54 (0.01–8.54)	2.91 (0.34–7.92)	.002
CA125, IU/mL	151.5 (13.0–809.1)	182.0 (29.2–1388)	.752
Infertility duration (yr)	5.0 (0–16)	3.0 (0–11)	.119
Preoperative uterine volume, cm^3^	353.67 (93.0–2097.7)	327.76 (134.4–743.6)	.262
Nodule weight, g	123.5 (3.0–320.0)	90.0 (3.0–240.0)	.333
Operative time, min	220.0 (68.0–440.0)	175.0 (65.0–305.0)	.048
EBL, mL	750 (100–2500)	500 (50–2000)	.016
Surgical platform			.977
Robot (n)	5 (17.9%)	2 (13.3%)	
Laparoscopy (n)	7 (25.0%)	5 (33.3%)	
Laparotomy (n)	16 (57.1%)	8 (53.3%)	
Endometrium distortion (n)	10 (35.7%)	8 (53.3%)	.264
Adenomyosis type			.073
Focal (n)	4 (14.3%)	6 (40.0%)	
Diffuse (n)	24 (85.7%)	9 (60.0%)	
Transfusion (n)	19 (67.9%)	6 (40.0%)	.078
Combined disease			
Endometriosis (n)	8 (28.6%)	8 (53.3%)	.109
Combined leiomyoma (n)	12 (42.9%)	6 (40.0%)	.856
Size of myoma, cm	2.5 (1.0–5.9)	2.2 (1.0–4.9)	.553
Number of myoma	2 (1–4)	2 (1–3)	.892
Endometriosis size, cm	4.6 (1.0–10.0)	3.5 (1.0–8.5)	.755
ASRM stage	4 (1–4)	4 (3–4)	.755
IVF history (n)	25 (89.3%)	12 (80.0%)	1.00
The number of pregnancy trial after surgery	2 (0–5)	1 (0–4)	.848
Follow-up duration, mo	18.5 (3–42)	12.0 (6–38)	.256

Table 2 shows the pregnancy outcomes. There were 15 cases of successful pregnancy. The pregnancy success rate was 34.9%. One case of pregnancy was through natural conception while 14 were through in vitro fertilization and embryo transfer. Two ectopic pregnancies were not regarded as successful pregnancies.

**Table 2:** Pregnancy outcomes.

Parameter	n.
Pregnancy success	15
Natural pregnancy	1
IVF-ET	14
Miscarriage	3
Live birth	3
Preterm labor	2

Table 3 shows independent factors affecting pregnancy success. Age (odds ratio [OR]: 0.210, 95% confidence interval [CI]: 0.046–0.956, *P* = .044) and the AMH level (OR: 6.076; 95% CI: 1.259–29.332, *P* = .025) were associated with pregnancy success in the multivariate analysis. Additionally, patients were divided into two groups according to age, AMH, type of adenomyosis, and the presence of endometrial distortion to compare the number of successful pregnancies among the total number of pregnancy trials (Table 4). Table 5 summarizes the delivery data.

**Table 3:** Univariate and multivariate logistic regression analysis for predicting pregnancy success after adenomyomectomy.

	Univariate analysis		Multivariate analysis	
Clinical factors	OR (95% CI)	*p*	OR (95% CI)	*p*
Pregnancy success				
Age (≥38^∗^)	0.241 (0.050–1.172)	.078	0.210 (0.046–0.956)	.044
AMH (≥1.18^∗^)	6.440 (1.221–33.963)	.028	6.076 (1.259–29.332)	.025
Operative time (≥205.0min^∗^)	0.736 (0.108–4.990)	.753		
EBL (≥500ml^∗^)	1.027 (0.145–7.266)	.979		
Adenomyosis type				
Focal	1			
Diffuse	0.362 (0.055–2.391)	.292		

**Table 4:** The number of pregnancy success per total number of pregnancy trial with divided group according to clinical factors.

Clinical factors	n.	The number of pregnancy success per total number of pregnancy trial (%)
Age		
≥38^∗^	19	4/35 (11.4)
<38^∗^	24	11/28 (39.3)
AMH		
≥1.18^∗^	22	12/36 (33.3)
<1.18^∗^	21	3/27 (11.1)
Adenomyosis type		
Focal	10	6/12 (50.0)
Diffuse	33	9/51 (17.6)
Endometrium distortion		
No	25	7/38 (18.4)
Yes	18	8/29 (27.6)

**Table 5:** Delivery data.

Case n.	Age	Delivery type	Birth weight	GA at delivery	EBL at delivery	Complications	Others
1	37	Elective C/S	3050g	38w+0	800	IIOC Preterm labor	Mcdonald operation at GA 22w+6
2	37	Elective C/S	2670g	36w+4	1200	One fetal death (DCDA twin) at GA 12w+0	
3	37	Elective C/S	3120g	38w+6	500	IIOC	Mcdonald operation at GA 17w+4

## 4 Discussion

Adenomyomectomy is a surgically challenging procedure.^[17]^ It is difficult to distinguish the affected adenomyotic tissue from the normal myometrium.^[18]^ In addition, proper uterine reconstruction to minimize the dead space and secure hemostasis is a difficult task.^[16]^ Due to these surgical difficulties, adenomyomectomy is performed after careful consideration. In the present study, we determined the most appropriate candidates for adenomyomectomy who desired pregnancy. We also analyzed the data regarding fertility outcomes after adenomyomectomy.

Saremi et al performed abdominal adenomyomectomy with wedge-shaped removal of adenomyotic tissue after sagittal uterine incision in 103 patients with adenomyosis. They reported a pregnancy rate of 30% among 70 patients who attempted pregnancy. Sixteen of 70 patients reached full-term live birth.^[19,20]^ Kishi et al^[9]^ reported a pregnancy rate of 31.4% (32/104) after laparoscopic adenomyomectomy while Osada et al^[20]^ reported 61.5% (16/26). In the present study, the pregnancy rate was 34.9% (15/43) after adenomyomectomy, consistent with previously reported fertility outcomes in other studies. The lower pregnancy rate in our study compared to the study by Osada et al^[20]^ may be attributed to the fact that our patients were diagnosed with infertility preoperatively.

Unlike the aforementioned studies, we performed adenomyomectomy using various surgical platforms, including laparotomy, laparoscopy, and robotic platform. In our previous study, surgical outcomes of robotic adenomyomectomy were comparable to those of laparoscopic adenomyomectomy.^[16]^ We had speculated if robotic adenomyomectomy showed similar fertility outcomes as other platforms. Particularly, there have been concerns that complete excision of adenomyotic lesions is highly difficult with a laparoscopic or robotic platform because of the absence of tactile sensations.^[21]^ However, the surgical platform was not associated with pregnancy success in our study. To date, selecting the surgical platform has not been an important factor for pregnancy success. Randomized trials with larger sample sizes are required in the future to confirm this issue. Additionally, pregnancy complications, such as uterine rupture, should be evaluated in women who have undergone robotic adenomyomectomy.

Our results showed that age and the AMH level might be predictive factors for pregnancy success after adenomyomectomy. This finding is consistent with the results reported by Kishi et al^[9]^ for 102 women desiring pregnancy who had undergone laparoscopic adenomyomectomy. They reported pregnancy rates of 41.3% and 3.7% in women aged ≤ 39 and ≥ 40 years, respectively.^[9]^ The authors concluded that adenomyomectomy is a beneficial treatment for women aged ≤ 39 years.^[9]^ Similarly, in the present study, a relationship between age and pregnancy success was suggested, and only two (2/15, 13.3%) women aged ≥ 40 years had successful pregnancy. Our study revealed that the AMH level was an independent predictive factor for pregnancy success in the multivariate analysis. Younger age and the AMH level reflect ovarian function. Therefore, clinicians and patients should consider ovarian reserve a predictive marker for successful pregnancy after adenomyomectomy.

The effect of myoma on fertility impairment depends on the tumor location.^[22]^ Intramural and submucosal myomas significant impact fertility.^[22,23]^ In a systematic review, Klatsky et al demonstrated that myoma distorting the uterine cavity was associated with a lower rate of implantation.^[24]^ Similarly, we hypothesized that adenomyotic lesion distorting the uterine cavity may be associated with a lower pregnancy rate. We categorized patients into two groups according to the presence/absence of distortion of the endometrium line. Our results revealed that endometrial distortion due to adenomyosis did not affect pregnancy success.

Instead of the distorting effect, the type of adenomyosis seemed to be relevant to the pregnancy outcome. Although the difference approached significance, patients with focal adenomyosis showed a higher rate of pregnancy success compared to those with diffuse adenomyosis (40.0% vs 14.3%, *P* = .073). Operative time was shorter while operative blood loss lower in the pregnancy success group. This observation might be related to the type of adenomyosis, as focal lesions are easier to operate on compared to diffuse lesions. Moreover, the number of successful pregnancies among the total number of pregnancy trials was much higher in the focal group than in the diffuse group (50% vs 17.6%; Table 4). Hence, we postulated that the type of adenomyosis (focal type) might be positively correlated with pregnancy success. Larger studies are required in the future to validate these findings.

Similar to other retrospective studies, the present study had some limitations. The sample size may have been insufficient to fully elucidate factors related to pregnancy success. However, since adenomyomectomy is rarely performed, the sample size of the present study may not be small. We could not sufficiently evaluate pregnancy complications related to adenomyomectomy, including uterine rupture or preterm delivery. Uterine ruptures after adenomyomectomy have been previously reported.^[25]^ Since most patients returned to their hometown for antenatal care after confirmation of pregnancy, we could observe only three deliveries and not fully evaluate the adverse events associated with adenomyomectomy during pregnancy. Therefore, we are currently planning a prospective study to evaluate pregnancy outcomes. All surgeries were performed by different surgeons using various surgical platforms. We believe that surgeons’ proficiency did not affect fertility outcomes, as all surgeons at our institution performed adenomyomectomy using the same method.

Nevertheless, our study contributes significantly to the literature, as indications or fertility outcomes of adenomyomectomy have not been well established. Our study provides clinicians and patients with practical information regarding factors related to pregnancy success after adenomyomectomy.

In conclusion, ovarian reserve (age and AMH) and disease severity might be predictive factors for successful pregnancy in patients who have undergone adenomyomectomy. Adenomyomectomy should be considered for women desiring pregnancy and having appropriate ovarian reserve. Moreover, the pregnancy success rate of approximately 30% is anticipated after adenomyomectomy. This information will be beneficial for patients and clinicians before deciding on adenomyomectomy. Larger prospective studies are required to confirm our findings.

## Author contributions

**Conceptualization:** Yong Wook Jung.

**Data curation:** Seyeon Won, Ji Young Hwang, Yong Wook Jung.

**Funding acquisition:** Yong Wook Jung.

**Formal analysis:** Ji Young Hwang, Yong Wook Jung.

**Investigation:** Nara Lee, Miseon Kim, Mi Kyoung Kim, Mi-La Kim, Bo Seong Yun.

**Methodology:** Nara Lee, Miseon Kim, Mi Kyoung Kim, Mi-La Kim, Bo Seong Yun.

**Supervision:** Seok Ju Seong.

**Writing – original draft:** Seyeon Won.

**Writing – review & editing:** Seyeon Won, Seok Ju Seong.
